# The Damaging Effects of Perceived Crocodile Tears for a Crier’s Image

**DOI:** 10.3389/fpsyg.2020.00172

**Published:** 2020-02-18

**Authors:** Inge van Roeyen, Madelon M. E. Riem, Marko Toncic, Ad J. J. M. Vingerhoets

**Affiliations:** ^1^Center of Research on Psychological and Somatic Disorders, Department of Medical and Clinical Psychology, Tilburg University, Tilburg, Netherlands; ^2^Clinical Child and Family Studies, VU University Amsterdam, Amsterdam, Netherlands; ^3^Department of Psychology, University of Rijeka, Rijeka, Croatia

**Keywords:** crying, tears, genuine, image, perception

## Abstract

Emotional tears are uniquely human and play an essential role in the communication of distress in adults. Several studies have shown that individuals are more willing to offer emotional support and help a person in tears. Preliminary evidence suggests that this greater willingness to provide support is mediated via perceived warmth and helplessness. Moreover, tearful individuals are regarded as more reliable and honest. In the current study, we examined whether people can reliably distinguish genuine and fake crying, and what the consequences for the further evaluation of the crier are. A total of 202 participants (73 men, 129 women) were exposed to brief movie clips of genuine and fake crying adults and were asked to assess the criers. Results show that women were slightly better at identifying fake and genuine crying. How the crying was perceived subsequently seemed to have a strong influence on the further evaluation of the “crier.” Criers qualified as pretenders were perceived as significantly more manipulative, less reliable, less warm, and less competent. Further, the respondents felt less connected with the perceived pretenders, who also were less welcomed as friends, colleagues, neighbors, and babysitter. They were additionally qualified as significantly less fit for “reliable” professions (judge, teacher, police officer, scientist, and physician). In contrast, the ratings of their fitness for “unreliable” professions (banker, CEO, journalist, real estate salesman, and politician) yielded a significant difference in only one video clip (and contrary to expectations). Our findings thus indicate that the subjective labeling of crying as fake is associated with a significantly less positive perception of the “crying” person, regardless of whether the crying is actually fake or genuine. The qualification of tears as crocodile tears thus seems to affect the crier’s image strongly negatively.

## Introduction

*……tears are shed in the greatest quantity by people with the best characters* Petitus (1661)

Tearful crying is a uniquely human reaction to a wide variety of situations and stressors, including separation, loss, physical pain, and situations typically associated with feelings of helplessness as well as seeming positive situations such as weddings, proposals, victory, reunion, and exceptional achievements (Vingerhoets, 2013; Gračanin et al., 2018). Remarkably, despite the current high interest of researchers in emotions, the study of emotional tears has received just modest attention of the scientific community. Moreover, the functions of emotional tears were for a considerable period more subject of speculation of clinicians than the object of more systematic studies. Ever since Freud and Breuer (1995) launched their catharsis concept, the idea dominates that the function of emotional tears must be searched for in the crying individual him or herself. In other words, the focus was mainly on how crying impacted the well-being of the crier. Not only in the clinical literature but also in the lay literature, this conviction acquired a dominant position (Cornelius, 1986). Also, today, one can read popular articles and watch YouTube videos voicing the notion that crying brings relief and is healthy. Occasionally, even rather strong statements like “Cry or die” are utilized.

However, tearful crying also seems to serve essential communicative, interpersonal functions (Kottler, 1996; Nelson, 2005; see Gračanin et al., 2018, for a review). There is currently increasing evidence that emotional tears not only inhibit aggression (Hasson, 2009), and promote empathy in others, they also facilitate the willingness to connect and to provide help and succor (Vingerhoets et al., 2016). Moreover, Balsters et al. (2013), examining the influence of tears on the identification of sadness and the perceived need for social support, found that sadness was faster identified when tears were added to sad adult faces. Also, the perceived need for social support was greater when faces contained tears. Another study showed that observers are more willing to provide emotional support and tend to express less negative affect toward a crying than a non-crying individual (Hendriks et al., 2008).

Moreover, whereas in the popular media crying is often associated with weakness and a lack of competence, recent evidence suggests that criers are also seen as notably warmer (Van de Ven et al., 2017), and more reliable and honest (Píco et al., 2020). Currently, we do not know which conditions determine the reaction of the observers to a crying individual. Vingerhoets (2013) has formulated a preliminary model, in which factors like the characteristics of the crier (e.g., gender, status) as well of the observer (e.g., empathy, psychopathy), their mutual relationship (e.g., mother-child; romantic partners; therapist-client; chief – employee), the perceived appropriateness, and how the crier weeps (just moist eyes or uncontrolled crying) all might exert their influence. However, there is currently no research that has specifically addressed these factors.

Given these possible positive effects of tears on others, the display of this behavior may likely benefit those applying this strategy. Indeed, substantial anecdotal evidence and a few more systematic studies have addressed this issue (Buss et al., 1987; Vingerhoets and Bylsma, 2016). For example, narcissists (Alexander, 2003), highly neurotic women (Buss et al., 1987), and sociopaths (“the champions of the crocodile tears,” Stout, 2005) are known for their tactics of manipulation, including crying (Vingerhoets and Bylsma, 2016). Narcissistic crying has been qualified as “performed,” “inauthentic,” and “exploitative,” for instance, in therapeutic settings. This fake crying may trigger feelings of being controlled and devalued in therapists who observe the patient’s distress, but yet feel untouched by the whole experience. Crocodile tears may thus result in emotional detachment, and a lack of empathic connection, which contrasts with empathic feelings and sharing of distress experienced when witnessing genuine crying (Alexander, 2003).

Also in the courtroom, tears of defendants are frequently regarded as crocodile tears (Lefrevre, 2008; Glaberson, 2011). In these settings, what people consider as fake tears generally seems not to be appreciated, and a convict who is suspected of crying crocodile tears may be met with much disapproval and adverse reactions. Even defense attorneys have occasionally been accused of swaying juries with the power of tears to spare their client, appealing to the emotions of the jury instead of their reason (Lefrevre, 2008). The general implicit assumption thus seems to be that manipulating juries in the courtroom with crocodile tears may be beneficial for the defendant (Glaberson, 2011).

The detection of the truthfulness of others is an essential skill in everyday social interactions and legal settings. This raises the question of whether people can reliably distinguish between fake and real emotions, and, more specifically, between genuine and crocodile tears. The results of a few studies on deception do suggest that crocodile tears may be recognized and show that verbal and body language cues can reveal falsified sadness expressions, also in the case of false remorse (Porter and Yuille, 1995; Porter and Ten Brinke, 2008; Ten Brinke et al., 2012). More precisely, Ten Brinke et al. (2012) showed that, compared with genuine remorseful feelings, false remorse was accompanied by a broader range of emotions. This emotional turbulence may be reflected in the leakage of genuine, positive emotions during expressions of falsified sadness. Indeed, there is suggestive evidence that individuals show inconsistent emotional expressions during deception, possibly indicating that subtle emotional leakages in the face reveal an involuntary aspect of human behavior (Porter and Ten Brinke, 2008; Ten Brinke and Porter, 2012).

Do individuals or groups differ in the capacity to recognize fake expressions? Vrij and Mann (2001) examined the detection of real-life videotaped deception of relatives appealing for help concerning a missing family member whom they had murdered. Police officers, with an accuracy rate of 50%, did not outperform laypeople. Moreover, it has been shown that lie ability and lie production are positively related, indicating that, in particular, those who easily lie, are better at detecting deceitful others (Wright et al., 2013). Dark triad personality traits (narcissism, psychopathy, and Machiavellianism) have also been shown to predict the ability to detect deceitfulness, although associations may be sex-specific. Lyons et al. (2017) showed that narcissism was related to poor lie detection in women, possibly because of deficits in empathy, whereas Machiavellianism, a personality trait connected to manipulation of others, was a positive predictor of lie detection in men.

Concerning sex differences, Vrij and Mann (2001) reported higher accuracies in men than in women. However, a meta-analysis examining individual differences in the ability to detect deception based on 108 studies did not reveal substantial sex differences (Aamodt and Custer, 2006). There was also no evidence of associations with age or education. Similarly, a more recent meta-analysis concluded that individuals vary in their credibility when telling lies, but not in their ability to detect lies, suggesting that deception judgments depend more on the liar’s credibility than on any other individual difference factor (Bond and DePaulo, 2008).

One may, however, wonder if the ability to distinguish real and fake tears also depends on whether the crier employs surface acting or deep acting (Mesmer-Magnus et al., 2012). In the case of surface acting, it might be easier to recognize that the tears are not genuine than in the case of deep acting. In the latter case, actors memorize intense emotional episodes and re-experience the associated emotions. That implies that the produced tears are always real, although the associated emotion(s) that triggered the tears have no connection with the direct situation.

We know currently little about how the perception of tears as crocodile tears subsequently impacts the evaluation of the “crier.” Therefore, the primary objective of the current study was to examine how the perception of tears as real or fake subsequently impacts the evaluation of the crying individual. To that end, we exposed the participants to eight brief video-fragments of crying individuals. Four of them were real crying episodes, and the remaining four included acted crying episodes. We asked the participants for each video fragment to indicate whether it was genuine or acted crying.

Based on the literature showing emotional leakage during deception, one could expect that participants would be able to reliably recognize crocodile tears, although in the case of the use of very brief footages, without sound and possible deep acting, it might be unlikely that observers can make this distinction. We further asked the participants to rate the perceived reliability, warmth, tendency to manipulate, weakness, sincerity, and competence of the crying models and to indicate to what extent they felt connected with him or her. We additionally requested the participants to report how suitable the crying model was for a set of professions that are regarded as reliable (physician, judge, teacher, police officer, and scientist) and a set of professions deemed unreliable (journalist, banker, real estate agent, politician, and CEO) according to the Ipsos Mori Veracity Index, a survey that lists the most and least trusted professions (Ipsos MORI, 2016). Participants were also requested to evaluate the suitability of the crying person for different personal relationship roles (colleague, neighbor, friend, and baby sitter), in order to obtain an impression of the effects of this factor for everyday social life.

Given the suggestion that manipulative crying may result in emotional detachment and disapproval (Alexander, 2003), we further hypothesized that the labeling of tears as fake is associated with more negative qualifications of the “crier.” More specifically, we expected that those who are perceived as fake criers are evaluated as less reliable, warm, and sincere, and less suitable for reliable jobs and close relationships, but more manipulative and suitable for unreliable jobs. We also explored gender differences in the capacity to recognize crocodile tears, but, given the mixed findings in previous studies (Aamodt and Custer, 2006), we were not certain what we might expect.

## Materials and Methods

### Participants

The study was announced via social media. Although a considerable number of people showed interest, the final sample with complete data consisted of 202 participants (*N* = 129 women). The ages ranged from 16 to 70 years old (*M* = 31.15, *SD* = 15.05). There were no exclusion criteria. Power analysis using G^∗^Power 3.1 for paired *t*-test (the difference between two dependent means) showed that a sample size of 199 is sufficient to detect small effects [*d* = 0.2, α = 0.05, power 0.80, 2 groups (real and fake)]. Permission for this study was obtained from the local ethics committee, and all participants gave informed consent.

### Procedure and Measure

The participants were asked to complete an online survey during which they were exposed to eight brief video clips (without sound, duration between 2 and 7 s), depicting a real or fake crying individual. Video clips of fake criers originated from YouTube movies with actors (two men, two women), whereas video clips of real crying originated from YouTube movies showing genuinely crying individuals (two men, two women). The video clips were selected from a larger set of 66 videos from YouTube. The selection of the eight clips was based on the technical quality of the video, front view of the faces of the crying individuals, the clear display of rolling tears on the cheeks, and full visibility of the faces, necks, and shoulders.

The participants were not informed that tears of four of the criers were fake, and of four others were real. All participants viewed all eight video clips. After each video fragment, a set of questions was answered, addressing the perceived genuineness of the tears and the evaluation of the depicted crier. More precisely, participants were asked whether the depicted tears were real or fake and to indicate how confident (0–100%) they were about their answer. In addition, they were requested to evaluate the reliability, warmth, tendency to manipulate, weakness, sincerity, and competence of the crying model. With the Inclusion of Others in Self scale (Aron et al., 1992), we further assessed to what extent the participant felt connected with the depicted individuals. Finally, as a more indirect measure of perceived reliability and social attractiveness, and to obtain some clue to what extent the different ratings would translate to daily life, the participants indicated their enthusiasm to have the depicted individual in certain social roles in their private life (i.e., as a colleague, neighbor, friend, or babysitter), and to what extent they felt that the depicted individual was fit for a set of reliable and unreliable professions. More precisely, participants were asked to rate the perceived fitness of the depicted person for being a police officer, teacher, scientist, judge, and physician, professions that have previously been characterized as reliable according to the veracity index, and banker, real estate salesperson, CEO, politician, journalist, which are professions characterized as unreliable (Ipsos MORI, 2016). All ratings were conducted on VAS scales ranging from 1 to 100.

### Statistical Analysis

To evaluate whether the participants were able to distinguish between real and fake tears and to examine possible gender differences in the ability to detect crocodile tears, Chi-square tests were performed. To address the primary objective (i.e., to examine whether the perception of tears as real or fake impacts the further evaluation of the crying individual), a series of linear mixed-effect models were fitted. All data analyses were carried out within the R statistical environment (R Core Team, 2016). Preliminary analyses (factor and reliability analyses) were carried out with the help of package “psych” (Revelle, 2018), multilevel modeling was performed using package “lme4” (Bates et al., 2015), while visualizations were created with the aid of package “ggplot2” (Wickham, 2016).

The perceived role fitness (PRF) for the three different domains (private settings, reliable and unreliable jobs) were all measured with multiple items. In order to evaluate the appropriateness of computing a single indicator for the specified domains, a principal component factor analysis was conducted for each domain separately. A single component seemed to adequately represent the data, with approximately 70% of the items’ variance explained by the first component, regardless of the perceived role fitness domain) (Table 1).

**TABLE 1:** Principal components eigenvalues, reliabilities estimates, and mean inter-item correlation of perceived role fitness domains.

	*N* items	First component Eigenvalue	Cronbach’s α	Mean inter-item correlation
Private setting	4	2.79	0.85	0,59
Reliable jobs	5	3.18	0.86	0,54
Unreliable jobs	5	3.35	0.88	0,59

To test whether actual tears and perceived tears and their interaction have an impact on the dependent measures, individual judgments were modeled as a function of measurement type (perceived role fitness, warmth, manipulative tendency, reliability, weakness, sincerity, competence, and connectedness), actual tears (fake/genuine), perceived tears (fake/genuine) as well as their interactions. At the same time, we controlled for participants and video-clips as random effects in a series of hierarchical linear models. Categorical predictors (actual tears and perceived tears) were dummy coded with “fake” as the reference level for both of them. First, a null model (model 0) was estimated to assess the amount of between-person and between-clips judgment variance. The null model was used both to estimate the amount of variance that can be accounted for by individual differences (participants) and by manipulation (video clips) and also to serve as a null model to compare more complex models with. The addition of fixed parameters was evaluated in a stepwise fashion using the likelihood ratio test. Main effects of all variables were entered in model 1. Model 2 additionally included two-way interaction terms, while model 3 also included three-way interaction terms. As a significance indicator of every single parameter, bootstrap confidence intervals were computed (5000 samples). As a measure of overall effect size, Ω2 proposed by Xu (2003) was calculated and reported.

## Results

### Recognition of Genuine and Fake Tears and Gender Differences

Chi-square analyses of the contingencies of actual tears (genuine/fake) and perceived tears (genuine/fake) revealed that there is a statistically significant association between actual tears and perceived tears (χ^2^ = 33.61, *p* < 0.001). The diagonal elements in the total sample part of Table 2 are higher than the off-diagonal ones. On average, 57% of video clips were correctly categorized (significantly higher than chance). If we separate those contingencies by gender, it can be seen that there is no formally statistically significant association of actual and perceived tears in the male subsample (χ^2^ = 3.34, *p* = 0.06), while the opposite is true for the female subsample (χ^2^ =33.89, *p* < 0.001). More precisely, the accuracy rate of the men was 54%, while the female accuracy rate was 59% (male and female subsample part of Table 2).

**TABLE 2:** Number of occurrences for every combination of actual and perceived tears for the total sample and for the male and female subsample separately.

	Total sample		Men		Women	
			
	Perceived tears		Perceived tears		Perceived tears	
				
Actual tears	Fake	Genuine	Fake	Genuine	Fake	Genuine
Fake	424	384	146	146	278	238
Real	307	501	123	169	184	332

### How the Perception of Tears as Genuine or Fake Impact the Evaluation of the “Crier”

Table 3 presents the descriptives of the evaluations of the genuine and fake criers. The null-model (model 0) fitted the data poorly (Table 4). The ICC was 0.13, mostly related to between-person differences, meaning that almost 13% of the judgment variance can be attributed to between-person effects while the amount of between-clips variance was neglectable (less then 1%). The addition of fixed main effects increased the model fit significantly, as did the inclusion of both two-way and three-way interactions (Table 4).

**TABLE 3:** Descriptives (mean and SD) of certainty, competence, reliability, warmth, weakness, connectedness, sincerity, manipulation, role fitness, reliable job, and unreliable job for genuine and acted tears (left) and perceived genuine and perceived fake tears (right).

	Actual tears				Perceived tears			
		
	Genuine		Fake		Genuine		Fake	
				
	*M*	*SD*	*M*	*SD*	*M*	*SD*	*M*	*SD*
Certainty	57.56	23.94	57.83	25.11	58.66	23.78	56.52	23.37
Competence	38.32	22.46	40.32	22.90	43.61	21.97	34.13	22.49
Reliability	43.30	25.04	40.87	24.80	54.93	21.49	26.53	19.35
Warmth	43.83	26.02	41.52	26.65	54.22	23.73	28.69	22.29
Weakness	32.34	24.73	30.76	24.75	28.08	23.22	35.75	25.88
Connectedness	22.85	25.16	22.62	25.72	34.03	26.27	9.06	15.90
Sincerity	45.05	26.97	40.12	26.42	58.05	22.09	23.87	18.92
Manipulative	34.44	26.40	37.95	27.29	24.06	19.84	50.88	27.01
Role fitness	44.42	22.92	42.11	22.17	52.28	20.82	32.35	19.61
Reliable job	30.35	19.81	34.37	19.78	37.17	19.80	26.53	18.40
Unreliable job	30.24	20.57	34.71	20.60	33.28	19.84	31.50	21.67

**TABLE 4:** Fit indices and significance testing of the fitted models.

	df	AIC	BIC	Ω^2^	log-likelihood	χ^2^	df(χ^2^)	*p*
Model 0	4	164023	164054	0.14	−82008			
Model 1	16	160740	160865	0.28	−80354	3307.270	12	<0.001
Model 2	37	157750	158038	0.39	−78838	3032.212	21	<0.001
Model 3	47	157748	158114	0.40	−78827	21.508	10	<0.05

The addition of three-way interaction terms had a modest (in terms of effect size estimates) but significant effect. Model 3 estimates, *t*-values, and 95% bootstrapped confidence intervals are presented in Table 5.

**TABLE 5:** Estimated coefficients, *t*-values, bootstrap 95% confidence intervals, and variance components of model 3.

	Estimate	*t*	95% C.I.	
			
Main effects			Lower-bound	Upper-bound
Intercept	58.44	50.37	55.99	60.83
Warmth	–28.79	–21.38	–31.42	–26.04
Manipulative tendency	–7.66	–5.69	–10.23	–4.90
Reliability	–31.50	–23.39	–34.19	–28.79
Weakness	–24.51	–18.20	–27.08	–21.81
Sincerity	–34.83	–25.86	–37.51	–32.07
Competence	–22.52	–16.73	–25.22	–19.83
PRF private settings	–25.41	–18.87	–28.04	–22.74
PRF reliable job	–29.39	–21.82	–31.98	–26.66
PRF unreliable job	–24.30	–18.05	–27.00	–21.56
Perceived connectedness	–24.30	–18.05	–27.00	–21.56
Perceived tears (real)	–1.30	–0.71	–4.12	1.49
Actual tears (real)	–4.00	–2.47	–7.23	–0.68
**Perceived tears 2-way interaction**				
Warmth	26.26	13.44	22.40	30.14
Manipulative tendency	–25.73	–13.17	–29.66	–21.93
Reliability	30.59	15.66	26.61	34.60
Weakness	–5.38	–2.76	–9.16	–1.51
Sincerity	36.02	18.44	32.09	39.81
Competence	10.56	5.40	6.63	14.53
PRF private settings	20.40	10.44	16.48	24.34
PRF reliable job	12.48	6.39	8.71	16.16
PRF unreliable job	2.49	1.27	–1.48	6.40
Perceived connectedness	28.53	14.60	24.61	32.47
**Actual tears 2-way interaction**				
Warmth	2.29	1.10	–1.92	6.29
Manipulative tendency	4.81	2.32	0.66	8.97
Reliability	3.58	1.72	–0.53	7.87
Weakness	8.90	4.29	4.54	12.97
Sincerity	5.20	2.50	0.93	9.37
Competence	0.32	0.15	–3.82	4.49
PRF private settings	2.96	1.43	–1.21	7.12
PRF reliable job	–1.41	–0.68	–5.61	2.66
PRF unreliable job	–1.70	–0.82	–5.89	2.50
Perceived connectedness	3.10	1.49	–1.09	7.26
Perceived tears	6.32	2.84	2.36	10.33
**Perceived/Actual tears 3-way interaction**				
Warmth	–5.67	–2.02	–11.19	–0.01
Manipulative tendency	–6.97	–2.49	–12.38	–1.36
Reliability	–8.57	–3.06	–14.08	–2.89
Weakness	–10.12	–3.61	–15.67	–4.54
Sincerity	–8.40	–3.00	–13.77	–2.65
Competence	–5.76	–2.06	–11.36	–0.05
PRF private settings	–5.38	–1.92	–11.12	0.26
PRF reliable job	–6.68	–2.38	–12.06	–1.07
PRF unreliable job	–4.59	–1.64	–10.14	1.12
Perceived connectedness	–10.87	–3.88	–16.44	–5.20

**Random effects**	**Variance**			

Subjects	78.09			
Video clips	0.73			
Residual	403.45			

*PRF, perceived role fitness.*

The observation that all the main effect estimates are statistically significant, except for the perceived genuine tears, means that every domain/variable is estimated significantly lower than the intercept value (58.44) in the actual false/perceived false tears condition. Those main effects are not of interest because they serve as a starting point for judgments in the actual fake/perceived fake condition. The perceived tears two-way interaction parameters show that the judgments of positive personality aspects (warmth, reliability, sincerity, competence, perceived role fitness in private settings, and reliable jobs) are higher in the perceived genuine condition than in the perceived false condition. The same is true, but in the opposite direction, for the perceived manipulative tendencies and weakness, while there are no differences for PRF for unreliable jobs. The actual tears two-way interactions appeared to have a more subtle effect. Fewer parameters are statistically significant. More precisely, only perceived weakness was evaluated as higher in the case of not correctly identified actual tears with manipulative tendencies and sincerity having a marginally significant difference. The three-way interaction shows a small effect. The model estimates are not significantly different or are slightly smaller than zero, meaning that the combination of genuine actual tears perceived as genuine does not significantly increase or change the estimates.

**FIGURE 1:**
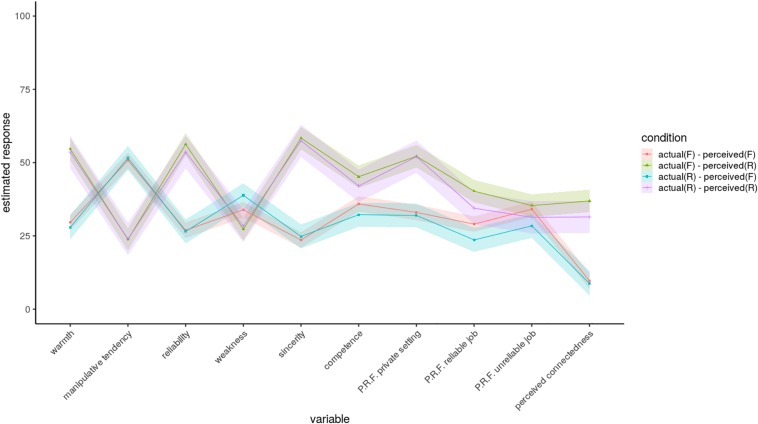
Estimated judgments by variable type and actual/perceived tears (model 3; confidence intervals presented as a ribbon). PRF, perceived role fitness.

If we take a look at the model’s prediction as a whole [the model’s estimated response for actual (fake/genuine) and perceived (fake/genuine) tears condition; Figure 1], it is clear that the perception of the tears as real, regardless of their actual nature, has a significant impact on almost all judgments. The same effect cannot be attributed to the actual tears in the presented clips. The sole perception of the tears as genuine evoked a more positive judgment of the depicted crying model in terms of higher perceived warmth, reliability, sincerity, competence, PRF in private settings and reliable jobs, and perceived connectedness and lower perceived manipulative tendency. The situation regarding the perceived weakness and the PRF for unreliable jobs appeared to be less clear than for the other domains. More specifically, we found no significant differences in PRF for unreliable jobs, while there were significant differences in perceived weakness, although the pattern that was observed in other domains appears dampened. Genuine tears, when perceived as fake, produced a marginally higher weakness estimate than when the tears were perceived as real, regardless of their actual nature.

## Discussion

The present study was specifically designed to examine how the perception of tears as genuine or false subsequently impacts the further evaluation of the “crier.” To that end, we exposed participants to brief movie fragments of genuine and fake crying adults and asked them to evaluate the depicted models. We found strong evidence that it is, in particular, the perception of the tears as genuine or fake, rather than their actual state, that determines how the “crier” is further qualified. Whereas the direct comparison of the genuine and fake criers yielded suggestive evidence that genuine criers were considered more reliable, these effects were rather small. In contrast, individuals, correctly or incorrectly, identified as genuine criers were rated as substantially more reliable, more welcome in different private roles, and more fit for reliable professions than those deemed as crying crocodile tears. Partly as expected, female participants slightly performed better than chance, although they still had considerable difficulty in determining whether a crying episode was genuine or fake. Also given the findings that the participants were not very certain about the correctness of their qualification of the tears as real or fake, we are reluctant to conclude that the current results add to previous studies showing that subtle emotional leakages can reveal falsified sadness (Ten Brinke et al., 2012).

Our findings strongly suggest that, despite the awareness of their uncertainty and relatively poor ability to distinguish between genuine and fake crying, the participants nevertheless seemed to attach much value to their judgment, which subsequently determines how they further perceived the “criers.” Those who were regarded as producing crocodile tears received much stronger negative and/or less positive qualifications than those who were considered as genuine criers. The former group were also less welcome in the private lives of the participants, and they were deemed less fit for what people generally regard as reliable professions. In contrast, the results of the fitness for unreliable professions yielded no clear differences. Our findings thus indicate that the subjective labeling of crying as fake is associated with a significantly less positive perception of the “crying” individual, regardless of whether the crying is indeed fake. Perceived crocodile tears thus have a damaging effect on the crier’s image.

A strength of the present study is that we exposed the participants to eight different crying individuals and that the results were very similar for all these different criers, indicating a high generalizability of the findings. However, the current study also suffers from some limitations. First, one should be aware that the exposure times to the stimuli were rather short, and there was no auditory information. Consequently, it was not possible to detect emotional leakage, as in the studies of Vrij and Mann (2001) and Ten Brinke et al. (2012). It is, therefore, plausible that these stimuli characteristics make the task of distinguishing between fake and real tears more difficult than it is in real life. Additionally, as outlined in the introduction, the recognition of tears as fake or genuine may also be particularly challenging if the actors apply deep acting strategies, meaning that the expression fits the internal feelings and in a certain sense even cannot be considered as fake.

Further, we implicitly assumed that the labeling of the crying as real or fake subsequently determined how the “crier” was perceived. However, it cannot be ruled out that the participants saw possibly cues in the physical appearance or demeanor of the targets that determined both the identification of the crier as real or fake and the further positive or negative qualifications. We, therefore, recommend that in future studies, the participants provide a first evaluation of the depicted criers in a neutral state several weeks before the ultimate test, allowing the researchers to explore whether the models in a neutral state perhaps show some signals that observers associate with negative or positive characteristics. When they subsequently rate them once more based on their crying, we can, with greater confidence, conclude that the perceived genuineness of the tears influences the further evaluation of the target. Alternatively, future studies should manipulate real and fake crying and present the movie clips with the same actor to participants in order to rule out influences of variation across videos, preferably during lab sessions. However, it should be noted that, because of the low variance of the dependent variables that can be attributed to between-person variations, the impact of movie diversity, even if uncontrolled for in the design, is neglectable. Since the participants completed the survey online at a place of their own choice, we also cannot rule out influences of external distractors. Note, however, that it is unlikely that the effects of crocodile tears will be weaker in well-controlled laboratory conditions when the participants’ attention to the stimuli is optimal. In contrast, it seems more plausible that the effects of crocodile tears may even be stronger in well-controlled laboratory conditions when participants’ attention to stimuli is optimized. Another limitation is that we cannot rule out that participants had seen the movie clips before, although this is unlikely because we presented clips of unknown actors from YouTube. Thus, an extensive replication is needed before we can draw more definite conclusions.

The present findings nevertheless suggest that crocodile tears likely are met with negative consequences. In that sense, this study yielded most relevant findings corroborating the anecdotal evidence about the negative consequences of fake crying in therapeutical (Alexander, 2003) and court settings (Lefrevre, 2008; Glaberson, 2011). Perhaps people implicitly feel that tears represent an honest signal and that misusing them for manipulation may not just be some minor transgression but rather a sign of intrinsic badness and lack of trustworthiness, sufficiently negative characteristics to warrant social rejection. The other side of the coin is that genuine crying seems associated with warmth, honesty, and reliability, characteristics that render an individual attractive for social exchange and collaboration (Gračanin et al., 2018). Our findings reveal that the participants felt more connected with those individuals who they perceive as real criers and were more willing to have them in certain roles in their private life. The present findings thus corroborate with previous findings demonstrating that tearful individuals, as compared with the same individuals without tears, are perceived as warmer, more reliable, and honest (Zickfeld et al., 2018; Píco et al., 2020). An important implication for crying research could be that it makes sense to check whether the participants perceive the tears as fake or genuine, because that might have substantial impact on the further evaluation. It seems that this is a factor that should be added to the preliminary, above discussed model of Vingerhoets (2013) on the possible relevant factors.

Once it has been established that genuine tearful individuals are perceived as warmer and more reliable, the next logical and intriguing step is to establish whether individuals who tend to cry more are actually morally superior to non-criers. A recent self-report study yielded some first evidence that that might indeed be the case. Vingerhoets et al. (2018) demonstrated a positive association between self-reported crying proneness and the self-reported tendency to display prosocial behavior. Moreover, those who reportedly tend to cry more often showed stronger disgust reactions to and disapproval of social transgressions of others. Future research needs to replicate and extend these observations, preferably with real prosocial behavior as dependent variables, rather than just self-report.

Interestingly, in particular in the popular literature, crying is predominantly associated with a variety of negative connotations (e.g., weak, not competent, emotionally not stable, manipulative). However, the current study yielded first data indicating that those who genuinely cry are much appreciated and most welcome in our private lives. The problem, however, is that the tears have to be reliably perceived as genuine, which might be problematic because observers are not always very accurate in distinguishing genuine from fake tears.

## Data Availability Statement

The raw data supporting the conclusions of this article will be made available by the authors, without undue reservation, to any qualified researcher.

## Ethics Statement

The studies involving human participants were reviewed and approved by the Ethics Review Board (ERB) of the School of Social and Behavioral Sciences of Tilburg University. The patients/participants provided their written informed consent to participate in this study.

## Author Contributions

IR collected the data. IR, MR, and AV wrote the manuscript. MT analyzed the data, wrote the results section, and commented on the manuscript.

## Conflict of Interest

The authors declare that the research was conducted in the absence of any commercial or financial relationships that could be construed as a potential conflict of interest.
